# Sex/Gender Beliefs are Strongly Related to Either Right-Wing Authoritarian Conventionalism or Left-Wing Authoritarian Anti-Conventionalism

**DOI:** 10.1007/s10508-025-03371-4

**Published:** 2026-02-18

**Authors:** Alex Bertrams, Ann Krispenz

**Affiliations:** Division of Educational Psychology, Institute of Educational Science, University of Bern, Fabrikstrasse 8, 3012 Bern, Switzerland

**Keywords:** Anti-conventionalism, Conventionalism, Gender, Left-wing authoritarianism, Right-wing authoritarianism, Sex

## Abstract

Previous research investigating the correlates of sex/gender beliefs (i.e., “Sex/gender is binary and permanently fixed” vs. “Sex/gender is nonbinary or changeable”) focused on right-wing authoritarianism (RWA). In the present research, we aimed at conceptually replicating previous findings on the relationship between sex/gender beliefs and RWA conventionalism. As a novel aspect, we additionally investigated the association between sex/gender beliefs and left-wing authoritarianism (LWA) anti-conventionalism. In two preregistered studies, sociodemographic diverse samples from the USA (*N* = 1431) and the UK (*N* = 1390) completed measures of sex/gender beliefs, RWA (and social dominance orientation in Study 2), and LWA. In line with our expectations, in both studies we found that the binary/fixed sex/gender belief was strongly associated with RWA conventionalism while the nonbinary/changeable sex/gender belief was strongly associated with LWA anti-conventionalism. Thus, in both studies, opposing sex/gender beliefs were related to rigid and restrictive authoritarianism, but to different ideological sides. Our results indicate that one-sided arguments about (right-wing) authoritarianism and specific sex/gender beliefs are shortsighted.

**Supplementary Information:**

The online version contains supplementary material available at 10.1007/s10508-025-03371-4.

## Introduction

In recent years, different conceptualizations of sex and gender have become a contentious issue in education, science, media, politics, and the general public (e.g., Ainsworth, 2015; De France et al., 2025; Del Giudice, 2023; Elias & Colvin, 2020; Gillig et al., 2018; Kanamori et al., 2017; Lim et al., 2023; Maksymov, 2024; Meyer, 2022; Schudson & Morgenroth, 2022). In the controversy, the two terms “sex” and “gender” are roughly distinguished as follows: On the one hand, the term sex is typically used to refer to a biological definition based on anatomy and physiology (i.e., being either male or female); however, in this regard, the term intersex must also be mentioned, which describes individuals with any combination of chromosomes, anatomy, gonads, hormones, and/or secondary sex characteristics that does not fit into the binary male or female biological sexes (Astle et al., 2024; Blackless et al., 2000). On the other hand, the term gender is used to refer to a sociopsychological definition based on self-determination (as a man, a woman, nonbinary, genderqueer, genderfluid, etc.) which does not necessarily need to be identical with one’s biological sex (Ching & Xu, 2018; Elias & Colvin, 2020; National Academies of Sciences, Engineering, and Medicine, 2022). More specifically, the National Academies of Sciences, Engineering, and Medicine (2022) define sex as “a multidimensional construct based on a cluster of anatomical and physiological traits that include external genitalia, secondary sex characteristics, gonads, chromosomes, and hormones” and gender as “a multidimensional construct that links gender identity, which is a core element of a person’s individual identity; gender expression, which is how a person signals their gender to others through their behavior and appearance (such as hair style and clothing); and cultural expectations about social status, characteristics, and behavior that are associated with sex traits.” Other institutions, for instance Statistics Canada (2024), apply very similar definitions. The differentiation between sex and gender becomes particularly relevant when individuals’ specific sex/gender beliefs lead to opposing ideological views (e.g., about whether people can officially choose their own gender or not; Kanamori et al., 2017).

The present research focuses on one particular aspect of this controversy—the association between agreeing with a specific sex/gender belief (for the terminology “sex/gender belief,” see Kanamori et al., 2017) and holding authoritarian attitudes. In studies investigating the relationship between sex/gender beliefs and authoritarianism, it is assumed that authoritarianism contributes to the examined sex/gender beliefs (e.g., Perez-Arche & Miller, 2021). Until now, however, there have exclusively been studies investigating this question with regard to right-wing authoritarianism (RWA), finding the traditional or conventional sex/gender belief (i.e., “Sex/gender is binary and permanently fixed”) to be associated with RWA (e.g., Ching et al., 2020; Makwana et al., 2018; Perez-Arche & Miller, 2021; Schudson & van Anders, 2022). The aim of the present research is to replicate these findings but to extend the existing literature for a more balanced scientific perspective. To this end and for the first time, we examined the relationship between the non-conventional sex/gender belief (i.e., “Sex/gender is nonbinary or changeable”) and left-wing authoritarianism (LWA).

### Fundamentally Opposing Sex/Gender Beliefs

From a strictly traditional or conventional perspective, the sex and gender of the *homo sapiens* is binary; that is, there is a female and a male biological sex to which (almost) all individuals of the species can be assigned based on their germ cells, sex chromosomes, and sex organs (Arboleda et al., 2014; Perez-Arche & Miller, 2021). In this view, statistically rare deviations of the binary phenotype such as intersex individuals are regarded as anomalies (Arboleda et al., 2014; Danon & Sachs, 1957) in the context of the binarity. A strictly traditional view is also typically linked to the idea that sex and even gender are permanent and, thus, cannot readily be changed (Ching & Xu, 2018; Kanamori et al., 2017). This view also implies that changes of sexual characteristics (e.g., through hormone treatments, surgery, clothing, altering of one’s tone of voice, and further non-verbal behaviors) do not lead to an actual change of gender. In this binary view, an individual who changes their gender through self-determination (and complementary medical measures) from a man to a women would still be considered a man or at most a transgender woman but not a “real” woman. In sum, the strictly traditional view implies two crucial main beliefs: First, there are only two sexes (i.e., male and female) that are associated with the respective genders (i.e., man and woman) which implies a sex/gender binarity, and second, it is impossible to transition from one gender to the other (or even something beyond these two), that is, gender is permanent and fixed.

In contrast, the progressive or unconventional perspective disregards the biological conditions of sex as determining gender but instead emphasizes individuals’ gender identity (i.e., how they feel or experience to be): “Gender identity—often referred to as gender—is a person’s perception of who they are as male [a man], female [a women], both or neither” (Elias & Colvin, 2020, p. 210). The progressive perspective often characterizes the traditional binary view of sex and gender as a social and oppressive construct which needs to be deconstructed (cf. Butler, 1990). Therefore, at the core of the progressive sex/gender belief are two main thoughts: First, there are not only two but a variety of genders (Monro, 2005; Ridgeway & Saperstein, 2024). Associated with this first belief are gender-related self-descriptions, such as being nonbinary, genderqueer, or genderfluid. Second, anyone can alter their gender by respective self-determination independently of their biological attributes (Monro, 2005; Szydlowski, 2016). Thus, according to this belief, gender is not permanent or fixed. Based on the progressive perspective, transgender people can also change their gender from being a man to being a woman or vice versa.

Concerning the two sex/gender beliefs just presented, it should be considered that they do not have to be understood as dichotomies of crucial aspects of sex/gender beliefs (e.g., whether there are only or not only two genders; cf. Gilbert, 2009; Konopka et al., 2020). This means that these two beliefs do not necessarily exist in absolute terms or distinct categories, but that intermediate shades are possible. In line with this notion, in current research the degree of agreement between the two opposing sex/gender beliefs is measured on a continuum (e.g., Kanamori et al., 2017; Perez-Arche & Miller, 2021). Since arguments can be found for and against both a dichotomous conception and that of a spectrum of sex/gender beliefs, we have empirically considered both approaches in our present research.

### Authoritarianism

There is a long tradition of research on RWA (Adorno et al., 1950; Altemeyer, 1981, 2006; Saunders & Ngo, 2020). After some criticism of the initial conceptualizations, a more recent and frequently applied conceptualization of RWA takes a tripartite approach (Duckitt et al., 2010) according to which RWA is defined by three interrelated aspects: The most relevant RWA aspect for the present research is named conventionalism or traditionalism. Individuals high in RWA conventionalism tend to endorse traditional values and moral standards and combine these with the notion that everyone’s lifestyle within society should comply with their values and standards; thus, RWA conventionalism ingrains rigidity regarding the appropriate way to live in a given society. For instance, RWA conventionalism implies the view that “God’s laws” (e.g., regarding marriage or sexuality) must be strictly followed, while progressive values are considered immoral. A second RWA aspect is authoritarian submission—the uncritical endorsement and submission to established societal authorities, complemented by the view that everyone else in society should think and act the same way. Finally, RWA includes authoritarian aggression. This RWA aspect refers to the view that troublemakers and lawbreakers should be harshly punished by the established authorities to preserve law and order. In addition to the three RWA aspects, social dominance orientation (SDO) has been investigated as a complementary concept to RWA (e.g., Ho et al., 2015). SDO is defined as the preference for group-based hierarchy and inequality, and it has been conceptualized to reflect authoritarian leadership. Hence, SDO is a crucial addendum for RWA research, as RWA has been found referring to (only) authoritarian followership (Altemeyer, 2006). Therefore, while SDO is related to RWA, it is also different from RWA as it constitutes a fundamentally different construct (Duckitt, 2001, 2006).

In comparison to RWA, there has been less research on LWA although several attempts were made (e.g., Altemeyer, 1996; for an overview, see Costello et al., 2022; Nilsson, 2024); however, research into LWA has recently gained momentum. The conceptual and empirical work of Conway et al. (2018, 2023) and Costello et al. (2022) laid an important framework for understanding LWA. In the present study, we follow Costello et al.’s (2022) tripartite conceptualization and definition of LWA. As a first aspect, Costello et al. describe LWA anti-conventionalism as reflecting moral absolutism in terms of progressive values and a need for political homogeneity in one’s social environment. The authors also characterize this social attitude as being associated with the rejection of conservative values and conservative individuals as inherently immoral. As a second aspect of LWA, anti-hierarchical aggression refers to the notion that the perceived current societal structures and authorities need to be overthrown—if necessary also by means of violence. The third aspect of LWA is labeled top-down censorship which is the motivation to use authority to systematically and institutionalized suppress conventional, traditional, or otherwise politically right-wing opinions and behaviors. In the present study, in addition to RWA conventionalism, we investigated LWA anti-conventionalism as the second crucial aspect of authoritarianism. We chose Costello et al.’s approach because it describes anti-conventionalism as a clearly defined individual aspect of LWA that, at least grossly, mirrors RWA conventionalism. This will allow us to investigate the association between sex/gender beliefs not only with RWA conventionalism but also with LWA anti-conventionalism.

There is an ongoing controversy about the extent to which RWA (and SDO) and LWA are symmetrical, where they overlap, and where they differ from one another (e.g., Conway et al., 2018; Costello et al., 2022; Nilsson, 2024; Osborne et al., 2023). Although RWA and LWA undoubtedly diverge in terms of the political direction of the inherent values and attitudes (e.g., principally endorsing conventional, conservative, or traditional vs. anti-conventional, liberal, or progressive values; Osborne et al., 2023), there appear to be certain similarities. For instance, both RWA and LWA have been found to be related to dark-personality traits such as narcissism and psychopathy (Krispenz & Bertrams, 2024; Roy et al., 2021; Zeigler-Hill et al., 2021), dogmatism and different forms of prejudice such as racism and antisemitism (Allington et al., 2023; Conway et al., 2018, 2023; Fasce & Avendaño, 2023; Roy et al., 2021; Sibley & Duckitt, 2008), and the inclination to behave oppressively toward those who think differently, sometimes even violently (Costello et al., 2022; Osborne et al., 2023). For a recent comprehensive review of the differences and similarities between leftists and rightists in general, see Kteily and Brandt (2025).

In the present study, we followed Osborne et al.’s (2023) recently introduced integrative approach according to which, “at its core, authoritarianism entails the desire for group conformity at the expense of personal autonomy, accompanied by a deference to in-group authority figures and a desire to punish those who violate cherished in-group norms—regardless of whether these in-group norms reflect traditional or progressive values” (p. 221). Beyond this definition, however, there is an argument about the degree of symmetry between LWA and RWA in general. Thus, in our study, we examine whether crucial current conceptions of both RWA conventionalism and LWA anti-conventionalism are related to opposing sex/gender beliefs; however, due to the momentary state of research and debate (see Conway et al., 2018; Nilsson, 2024; Osborne et al., 2023), we cannot answer the basic conceptual question of how much the respective aspects of RWA and LWA are mirror images of each other. This also means that we cannot state that the RWA and LWA aspects are equivalent in the way that they can be conceptualized as the extreme poles on a common political spectrum. Therefore, our approach and findings will not directly be interpretable in the sense of a “horseshoe theory.”

For the variables of central interest in this study (i.e., RWA conventionalism and LWA anti-conventionalism), the currently available validated measures are respective subscales of the Authoritarianism-Conservatism-Traditionalism (ACT) Scales (Duckitt et al., 2010) and the LWA Index (Costello et al., 2022). What these two measures have in common is that they capture the degree of agreement with strong social-moral attitudes (cf. Krispenz et al., 2025) in their respective political spheres (conservative or progressive). On both sides, these attitudes are authoritarian in that they absolutize conservative or progressive moral values as the only inherently correct ones and, thus, that those attitudes should be imposed on everyone in society (e.g., “God’s laws about abortion, pornography, and marriage must be strictly followed before it is too late,” “It is important that we destroy the West's nationalist, imperialist values”). It is also authoritarian that divergent values and behaviors are not considered in the sense of attitudinal or lifestyle diversity but are absolutely rejected (e.g., “Trashy magazines and radical literature in our communities are poisoning the minds of our young people,” “The ‘old-fashioned ways’ and ‘old-fashioned values’ need to be abolished”). Consequently, individuals and lifestyles that represent different views, and are therefore not submissive to the moral view in question, are discredited (e.g., “The radical and sinful new ways of living and behaving of many young people may one day destroy our society,” “Conservatives are morally inferior to liberals”). Regarding these characteristics of authoritarianism which demand group conformity and undermine personal autonomy, in our view, RWA conventionalism and LWA anti-conventionalism conceptually mirror each other (for a principally different view, see Jost, 2024). It should be stressed, however, that the measures of RWA conventionalism and LWA anti-conventionalism were not developed as directly paralleling each other and, hence, differ in some ways such as in the individual contents and connotations of the concrete attitudes assessed (cf. Nilsson, 2024). For instance, the statements in the LWA anti-conventionalism measure by Costello et al. (2022) seem to use stronger language regarding those who think differently (e.g., “People are truly worried about terrorism should shift their focus to the nutjobs on the far-right,” “All political conservatives are fools”) than in the measure of RWA conventionalism by Duckitt et al. (2010). Thus, as stated above, we cannot assume directly comparable manifestations of authoritarianism in our study. Nevertheless, regarding RWA conventionalism and LWA anti-conventionalism, we basically assume that both concepts reflect authoritarian attitudes on opposing sides.

### Sex/Gender Beliefs and Authoritarianism

Studies on the debate around sex and gender issues typically focus on the beliefs of individuals high in RWA which are described as high in conventionalism (Altemeyer, 2006; Duckitt et al., 2010). Unsurprisingly, such studies typically find the conventional binary and permanently fixed sex/gender belief to be associated with RWA (e.g., Ching et al., 2020; Makwana et al., 2018; Perez-Arche & Miller, 2021; Schudson & van Anders, 2022). Based on these results, it has been argued that “by definition, those who endorse RWA want individuals to submit to authority and convention […] which TGNB [transgender and nonbinary] people are likely believed to be defying by not presenting or acting as their assigned gender” (Hatch et al., 2022; Perez-Arche & Miller, 2021, p. 184). Thus, it has been concluded that a rigid world view according to which society and its individuals must conform to time-honored traditions and social norms (i.e., RWA conventionalism) constitutes one important factor for the rejection of progressive gender views.

Therefore, in the present research, we assumed that the conventional belief of binary and permanently fixed sex/gender is related to RWA conventionalism as found in the above mentioned previous studies. This prediction can be logically derived from the definition of RWA conventionalism, as outlined by Perez-Arche and Miller (2021): Individuals high in RWA want everyone in society to collectivistically submit their lifestyle to conventional (i.e., traditional) values. As the belief of sex and gender as nonbinary/changeable is just the opposite of conventional or traditional, right-wing authoritarians should strongly reject it. In contrast, the relationship between sex/gender beliefs and the left-wing authoritarian side has not yet been empirically investigated. Based on the conceptualization of and empirical findings on LWA anti-conventionalism (Costello et al., 2022; Osborne et al., 2023), the same rationale as for RWA conventionalism in previous research (Perez-Arche & Miller, 2021) can inversely be applied to sex/gender beliefs: Just like individuals high in RWA conventionalism, individuals high in LWA anti-conventionalism want everyone in society to collectivistically bow to their morals (Osborne et al., 2023). In contrast to the conventional and conservative values in RWA, these values are anti-conventional and progressive in LWA. Thus, as the binary/fixed sex/gender belief is the conventional-conservative one, we secondly predicted that individuals with higher LWA anti-conventionalism should intensely oppose it and instead strongly advocate for the anti-conventional-progressive nonbinary/changeable sex/gender belief.

### The Present Research

In two preregistered studies, we empirically examined not only the association between the conventional-conservative binary/fixed sex/gender belief and RWA conventionalism, but also the association between the anti-conventional-progressive nonbinary/changeable sex/gender belief and LWA anti-conventionalism. While the first is a replication of existing findings (e.g., Ching et al., 2020; Makwana et al., 2018; Perez-Arche & Miller, 2021; Schudson & van Anders, 2022), the latter is intended to complete the picture with novel empirical findings. Our preregistered predictions were that a more pronounced binary/fixed sex/gender belief is related to higher RWA conventionalism, while a more pronounced nonbinary/changeable sex/gender belief should be related to higher LWA anti-conventionalism.

We consider RWA conventionalism and LWA anti-conventionalism to be the key aspects among the authoritarianism aspects for the present question. Independent of authoritarianism, the binary/fixed sex/gender belief and the nonbinary/changeable sex/gender belief may be direct logical emanations of general conventionalist and anti-conventionalist positions in terms of a specific issue; therefore, the sex/gender beliefs may also first and foremost be closely related to conventionalism and anti-conventionalism in individuals whose positions are extended into authoritarianism. Based on this thought, we also preregistered in Study 2 the following two hypotheses: First, the binary/fixed sex/gender belief is more strongly related to RWA conventionalism than to RWA authoritarian submission, RWA authoritarian aggression, and social dominance orientation. Second, the nonbinary/changeable sex/gender belief is more strongly related to LWA anti-conventionalism than to LWA anti-hierarchical aggression and LWA top-down censorship. These relationships between authoritarian (anti-)conventionalism and sex/gender beliefs are assumed to emerge over and above the relationships with sex/gender beliefs that the respective other authoritarian aspects may have.

Finally, we tested the (not preregistered) hypothesis that both predicted relationships (i.e., the binary/fixed sex/gender belief with RWA conventionalism and the nonbinary/changeable sex/gender belief with LWA anti-conventionalism) would hold over and above the potential influence of sociodemographic differences. This was done to rule out possible alternative explanations such as, for instance, an age effect: The nonbinary/changeable sex/gender belief has become more prevalent and integrated in the educational sector during the recent years (e.g., Mayo, 2021), and nowadays more young people than ever before report to identify as transgender (Jones, 2024); therefore, the nonbinary/changeable sex/gender belief could be related to younger age while, reversely, the binary/fixed sex/gender belief could be associated with older age. Furthermore, in previous research, RWA was found to be related to older age, while LWA was associated with younger age (Krispenz & Bertrams, 2024; Manson, 2020). Thus, the assumed relationships between sex/gender belief and the two authoritarianism aspects could result just from their respective overlap with age (i.e., an age effect) rather than from a specific authoritarian ideology.

In Study 1, we asked a sociodemographic diverse U.S. sample to complete standardized measures of RWA conventionalism, LWA anti-conventionalism, and sex/gender beliefs. In Study 2, we also measured the other RWA aspects (i.e., authoritarian submission, authoritarian aggression) and social dominance orientation as well as the other LWA aspects (i.e., anti-hierarchical aggression, top-down censorship) in a sociodemographic diverse UK sample as we included them into the analyses as covariates. Our hypotheses were tested by applying correlational, multiple regression, and logistic regression analyses.

## Study 1

### Method

#### Participants

To recruit a sociodemographic diverse U.S. sample, we used the research-oriented online data collection platform Prolific, where data quality has been found to be good (Douglas et al., 2023). In particular, we made use of Prolific’s option for recruiting the maximum deliverable nationally representative sample size which was 1500 participants at that time (see Costello et al., 2022, for more detail). The survey link was accessed 1530 times. Adhering to our preregistered criteria, we excluded *n* = 99 cases due to incomplete data (*n* = 32), duplicates (*n* = 16), and/or a failed attention check (*n* = 51). Thus, the final sample consisted of *N* = 1431 participants. Their detailed sociodemographic data are shown in Table S1 in the supplementary materials. Each participant was compensated with £0.90 (approx. USD 1.11 at that time).

#### Procedure and Measures

In addition to questions about sociodemographic data and the attention check, the participants completed two measures regarding their sex/gender beliefs, and a respective measure of RWA conventionalism and LWA anti-conventionalism. The measures of RWA conventionalism and LWA anti-conventionalism were presented in random order. Each measure was presented on a separate page.

*Attention Check*. The participants were explicitly instructed to omit answering the question who was the first president of the USA (Krispenz & Bertrams, 2024). Any provided answer was counted as failed attention check.

*Sex/Gender Belief*. We applied the unidimensional subscale “Sex/Gender Beliefs” of the Transgender Attitudes and Beliefs Scale (TABS; Kanamori et al., 2017) which contains 10 items tapping beliefs about how to view sex and gender in general (e.g., “Although most of humanity is male or female, there are also identities in between”), whether sex can be changed or re-interpreted (e.g., “If you are born male, nothing you do will change that;” inverse item), and how to interpret transgender and nonbinary gender identity (e.g., “A person who is not sure about being male or female is mentally ill;” inverse item). The items were responded to on seven-point scales from *strongly disagree* (1) to *strongly agree* (7). Corresponding to the approach of Kanamori et al. (2017), participants’ responses were coded such that higher scores represent a stronger belief in the nonbinary/changeable sex/gender view. Accordingly, lower scores reflect a stronger agreement with the binary/fixed sex/gender view.

As an additional measure of sex/gender beliefs, participants were asked: “In general, are there or are there not only two genders? Please indicate what you agree with most.” The two response options “In general, there are only two genders” (coded as 0) and “In general, there are not only two genders” (coded as 1) were presented in random order. Giving the coding, the value 0 corresponds to the binary/fixed sex/gender belief while the value 1 represents the nonbinary/changeable sex/gender belief.

*RWA Conventionalism*. We used the subscale traditionalism/conventionalism of the Authoritarianism-Conservatism-Traditionalism Scales (Duckitt et al., 2010). The twelve items (e.g., “The radical and sinful new ways of living and behaving of many young people may one day destroy our society”) were answered on seven-point scales from *strongly disagree* (1) to *strongly agree* (7). Higher scores indicate higher endorsement of RWA conventionalism.

*LWA Anti-Conventionalism*. The subscale anti-conventionalism of the LWA Index (Costello et al., 2022) was utilized. The 13 items (e.g., “Radical and progressive moral values can save our society”) were responded to on seven-point scales from *strongly disagree* (1) to *strongly agree* (7). The coding was such that the higher the scores the more agreement with LWA anti-conventionalism.

## Results

Table 1 depicts the descriptive statistics for the applied measures, including McDonald’s omega (ω) and the bivariate intercorrelations including the respective bias-corrected and accelerated bootstrap 95% confidence intervals (BCa 95% CI; throughout this article, each BCa 95% CI is based on 1000 bootstrap samples). Figure S1 in the supplementary material displays additionally the relationship between RWA conventionalism (Panel A) as well as LWA anti-conventionalism (Panel B) and sex/gender belief as measured with the TABS. In line with previous research and our expectation, the belief in nonbinary/changeable sex/gender as measured with the TABS (*r* =  − .75, BCa 95% CI [− .77, − .72], *p* < .001) as well as the single-item measure (*r* =  − .57, BCa 95% CI [− .60, − .54], *p* < .001) was negatively correlated with RWA conventionalism which means that the belief in binary/fixed sex/gender was positively associated with RWA conventionalism (cf. Kanamori et al., 2017). Vice versa, and also as predicted, the belief in nonbinary/changeable sex/gender as measured with the TABS (*r* = .66, BCa 95% CI [.63, .69], *p* < .001) as well as the single-item measure (*r* = .54, BCa 95% CI [.50, .58], *p* < .001) was positively correlated with LWA anti-conventionalism, which means that the belief in binary/fixed sex/gender was negatively related to LWA anti-conventionalism (cf. Kanamori et al., 2017).

**Table 1:** Results for Study 1 (U.S. sample)

Variable	ω	Possible range	Observed range	*M* (*SD*)	Correlations [BCa 95% CIs]		
					1	2	3
1. Sex/gender belief—TABS	.96	1.00–7.00	1.00–7.00	4.56 (1.71)	−		
2. Sex/gender belief—single item^a^	−	0 and 1	0 and 1	−	.78*** [.76, .79]	−	
3. RWA conventionalism	.94	1.00–7.00	1.00–7.00	3.24 (1.37)	− .75*** [− .77, − .72]	− .57*** [− .60, − .54]	−
4. LWA anti-conventionalism	.93	1.00–7.00	1.00–6.92	3.56 (1.32)	.66*** [.63, .69]	.54*** [.50, .58]	− .67*** [− .70, − .64]

*N* = 1431. BCa 95% CI = bias-corrected and accelerated bootstrap 95% confidence intervals based on 1000 bootstrap samples. ω = McDonald’s omega. TABS = Transgender Attitudes and Beliefs Scale. RWA = right-wing authoritarianism. LWA = left-wing authoritarianism. The total scores of the psychometric scales were obtained by averaging the responses to the scale items. For the TABS, lower values represent the stronger belief in binary/fixed sex/gender and higher values represent the stronger belief in nonbinary/changeable sex/gender (cf. Kanamori et al., 2017). For the single item on sex/gender belief, 0 represents the stronger belief in binary/fixed sex/gender and 1 represents the stronger belief in nonbinary/changeable sex/gender. Higher agreement with RWA conventionalism and LWA anti-conventionalism is represented by higher values of these variables. Pearson correlation coefficients are reported except for correlations with the dichotomous single item on sex/gender belief; for the latter point-biserial correlation coefficients are reported which are mathematically equivalent to the *t*-test (Rossi, 1989)

^a^The answer according to which in general, there are only two genders (coded = 0) was given by *n* = 811 participants (57%). The answer according to which in general, there are not only two genders (coded = 1) was given by *n* = 620 participants (43%)

*p < .05, **p < .01, ***p < .001. All *p*-values refer to two-tailed tests

To test whether the relationships between sex/gender belief and the two types of authoritarianism aspects are robust over and above sociodemographic differences, we additionally conducted multiple regression analyses for regressing sex/gender belief measured with the TABS and logistic regression analyses for regressing sex/gender belief measured with the single item. These additional analyses were not preregistered. We entered all sociodemographic variables collected (e.g., age, income, and political orientation; see Table S1) as covariates in the model regressing sex/gender beliefs on the respective authoritarianism variable (i.e., RWA conventionalism or LWA anti-conventionalism). Where necessary, we dummy-coded sociodemographic variables for this purpose (Tabachnick & Fidell, 2019). We found that the binary/fixed sex/gender belief as measured with the TABS (β =  − .53, *p* < .001) and the single item on sex/gender belief (*B* = 0.83, *p* < .001, odds ratio [*OR*] = 0.44) still was associated with RWA conventionalism when the sociodemographic variables were controlled for. Analogously, even when controlling the sociodemographic variables, the nonbinary/changeable sex/gender belief as measured with the TABS (β = .36, *p* < .001) and the single item on sex/gender belief (*B* = 0.62, *p* < .001, *OR* = 1.86) was still associated with LWA anti-conventionalism.

## Discussion

In accordance with our preregistered predictions, the binary/fixed sex/gender belief was more strongly agreed to by individuals higher in RWA conventionalism while the nonbinary/changeable sex/gender belief was more strongly agreed to by individuals higher in LWA anti-conventionalism. These relationships held over and above the participants’ sociodemographic differences. In Study 2, we aimed at replicating these findings in a UK sample. In addition, we sought first evidence that RWA conventionalism and LWA anti-conventionalism respectively (rather than the other RWA and LWA aspects) are the key ingredients in the relationship between sex/gender belief and authoritarianism.

## Study 2

### Method

#### Participants

For Study 2, data from a sociodemographic diverse UK sample was collected via Prolific. As in Study 1, Prolific’s option for recruiting a nationally representative sample size of 1500 participants was used. The survey link was accessed 1570 times. Adhering to the pre-registration criteria, we excluded *n* = 180 cases due to incomplete data (*n* = 56), duplicates (*n* = 30), and/or a failed attention check (*n* = 94). Thus, the final sample comprised *N* = 1390 participants. Their sociodemographic data are displayed in Table S2 in the supplementary materials. Each participant was compensated with £1.55 (approx. USD 1.98 at that time).

#### Procedure and Measures

The Authoritarianism-Conservatism-Traditionalism Scales, the Social Dominance Orientation_7_ Scale, and the LWA Index (see descriptions below) were presented in random order. The sociodemographic questions, each psychometric scale, and the single-item measure of sex/gender beliefs were presented on separate pages.

*Attention Check*. We applied the same attention check as in Study 1.

*Sex/Gender Beliefs, RWA Conventionalism, and LWA Anti-Conventionalism*. We applied the identical measures as in Study 1.

*RWA Authoritarian Submission and Authoritarian Aggression*. These variables were assessed with the respective subscales from the Authoritarianism-Conservatism-Traditionalism Scales (Duckitt et al., 2010). The twelve items for RWA authoritarian submission (e.g., “Our leaders should be obeyed without question”) and the twelve items for RWA authoritarian aggression (e.g., “We should smash all the negative elements that are causing trouble in our society”) were completed on seven-point scales from *strongly disagree* (1) to *strongly agree* (7). Higher scores indicate higher endorsement of RWA authoritarian submission and RWA authoritarian aggression.

*Social Dominance Orientation*. This variable was measured with the 16 items (e.g., “Some groups of people must be kept in their place”) of the Social Dominance Orientation_7_ Scale (Ho et al., 2015). The items were completed on seven-point response scales from *strongly oppose* (1) to *strongly favor* (7). Higher scores represent higher social dominance orientation.

*LWA Anti-Hierarchical Aggression and Top-Down Censorship*. We used the LWA Index (Costello et al., 2022) to measure LWA anti-hierarchical aggression (13 items; e.g., “Political violence can be constructive when it serves the cause of social justice”) and LWA top-down censorship (13 items; e.g., “We must line up behind strong leaders who have the will to stamp out prejudice and intolerance”) with the respective subscales. All items were answered on seven-point scales from *strongly disagree* (1) to *strongly agree* (7). The responses were coded such that higher scores indicate higher agreement with LWA anti-hierarchical aggression and LWA top-down censorship, respectively.

## Results

Table 2 displays the descriptive statistics and bivariate intercorrelations for the applied measures. See also Fig. S1 in the supplementary material for the relationship between RWA conventionalism (Panel C) as well as LWA anti-conventionalism (Panel D) and sex/gender belief as measured with the TABS. Analogously to Study 1, the nonbinary/changeable sex/gender belief as measured with the TABS (*r* =  − .59, BCa 95% CI [− .62, − .56], *p* < .001) as well as the single-item measure (*r* =  − .46, BCa 95% CI [− .50, − .42], *p* < .001) was negatively correlated with RWA conventionalism; that is, the binary/fixed sex/gender belief was positively associated with RWA conventionalism (cf. Kanamori et al., 2017). In addition, the nonbinary/changeable sex/gender belief as measured with the TABS (*r* = .49, BCa 95% CI [.44, .53], *p* < .001) as well as the single-item measure (*r* = .40, BCa 95% CI [.35, .45], *p* < .001) was positively correlated with LWA anti-conventionalism, meaning that the binary/fixed sex/gender belief was negatively related to LWA anti-conventionalism (cf. Kanamori et al., 2017). These results are in line with our predictions.

**Table 2:** Results for Study 2 (UK sample)

Variable	ω	Possible range	Observed range	*M* (*SD*)	Correlations [BCa 95% CIs]							
					1	2	3	4	5	6	7	8
1. Sex/gender belief – TABS	.94	1.00–7.00	1.00–7.00	4.33 (1.56)	−							
2. Sex/gender belief – single item^a^	−	0 and 1	0 and 1	−	.71*** [.69, .73]	−						
3. RWA conventionalism	.89	1.00–7.00	1.00–6.92	3.17 (1.05)	− .59*** [− .62, − .56]	− .46*** [− .50, − .42]	−					
4. RWA authoritarian submission	.93	1.00–7.00	1.00–6.83	3.49 (1.10)	− .43*** [− .48, − .37]	− .35*** [− .40, − .31]	.64*** [.60, .67]	−				
5. RWA authoritarian aggression	.93	1.00–7.00	1.00–7.00	4.15 (1.26)	− .56*** [− .60, − .52]	− .42*** [− .47, − .38]	.62*** [.58, .65]	.67*** [.64, .70]	−			
6. Social dominance orientation	.93	1.00–7.00	2.75–6.31	4.42 (0.44)	− .27*** [− .32, − .22]	− .24*** [− .28, − .19]	.31*** [.26, .35]	.30*** [.25, .34]	.36*** [.31, .40]	−		
7. LWA anti-conventionalism	.89	1.00–7.00	1.15–7.00	3.50 (1.06)	.49*** [.44, .53]	.40*** [.35, .45]	− .49*** [− .54, − .45]	− .47*** [− .51, − .43]	− .45*** [− .49, − .40]	− .24*** [− .29, − .19]	−	
8. LWA anti-hierarchical aggression	.90	1.00–7.00	1.00–6.85	2.66 (1.04)	.14*** [.09, .20]	.14*** [.08, .19]	− .16*** [− .22, − .11]	− .32*** [− .37, − .27]	− .17*** [− .23, − .11]	− .11*** [− .17, − .06]	.65*** [.62, .68]	−
9. LWA top-down censorship	.88	1.00–7.00	1.00–6.77	4.19 (1.05)	.25*** [.20, .30]	.16*** [.12, .21]	.02 [− .04, .08]	.14*** [.08, .20]	.07** [.02, .14]	− .01 [− .06, .04]	.42*** [.37, .46]	.20*** [.15, .26]

*N* = 1390. BCa 95% CI = bias-corrected and accelerated bootstrap 95% confidence intervals based on 1000 bootstrap samples. ω = McDonald’s omega. TABS = Transgender Attitudes and Beliefs Scale. RWA = right-wing authoritarianism. LWA = left-wing authoritarianism. The total scores of the psychometric scales were obtained by averaging the responses to the scale items. For the TABS, lower values represent the stronger belief in binary/fixed sex/gender and higher values represent the stronger belief in nonbinary/changeable sex/gender (cf. Kanamori et al., 2017). For the single item on sex/gender belief, 0 represents the stronger belief in binary/fixed sex/gender and 1 represents the stronger belief in nonbinary/changeable sex/gender. Higher agreement with the three RWA facets, social dominance orientation, and the three LWA facets is represented by higher values of these variables. Pearson correlation coefficients are reported except for correlations with the dichotomous single item on sex/gender belief; for the latter point-biserial correlation coefficients are reported which are mathematically equivalent to the *t*-test (Rossi, 1989)

^a^The answer according to which in general, there are only two genders (coded = 0) was given by *n* = 947 participants (68%). The answer according to which in general, there are not only two genders (coded = 1) was given by *n* = 443 participants (32%)

*p < .05, **p < .01, ***p < .001. All *p*-values refer to two-tailed tests

The bivariate intercorrelations (see Table 2) showed that the respective sex/gender beliefs were also related to the other RWA aspects, social dominance orientation, and LWA aspects: The binary/fixed sex/gender belief was associated with RWA authoritarian submission, RWA authoritarian aggression, and social dominance orientation while the nonbinary/changeable sex/gender belief was associated with LWA anti-hierarchical aggression and LWA top-down censorship.

In a preregistered multiple regression analysis, we regressed sex/gender belief as measured with the TABS on RWA conventionalism and additionally as covariates on RWA authoritarian submission, RWA authoritarian aggression, and social dominance orientation (for the results, see Table 3a). Corresponding to our assumption, the binary/fixed sex/gender belief was related to RWA conventionalism over and above the covariates in the model, and this relationship was also stronger compared to the ones with the covariates (i.e., the other aspects of right-wing authoritarian ideology). In addition, we regressed sex/gender belief as measured with the TABS on LWA anti-conventionalism and additionally as covariates on LWA anti-hierarchical aggression and LWA top-down censorship (for the results, see Table 3b). As expected, the nonbinary/changeable sex/gender belief was still related to LWA anti-conventionalism, and this relationship was stronger than the ones with the other LWA aspects. The binary/fixed sex/gender belief was also associated with RWA authoritarian aggression (see Table 3a), social dominance orientation (see Table 3a), and LWA anti-hierarchical aggression (see Table 3b) over and above the other variables in the multiple regression models. The nonbinary/changeable sex/gender belief was also associated with RWA authoritarian submission (see Table 3a) over and above the other variables in the multiple regression model. LWA top-down censorship (see Table 3b) was related to neither sex/gender belief in the multiple regression model.

**Table 3:** Multiple regression analysis regressing sex/gender beliefs (measured with TABS) in Study 2 (UK sample)

a) RWA and social dominance orientation	*B*	BCa 95% CI for *B*	*SE B*	β	*t*	*p*
Constant	8.37	[7.70, 9.03]	0.32	−	25.92	< .001
RWA conventionalism	− 0.62	[− 0.69, − 0.55]	0.04	− .42	− 14.56	< .001
RWA authoritarian submission	0.10	[0.01, 0.20]	0.04	.07	2.37	.02
RWA authoritarian aggression	− 0.41	[− 0.50, − 0.34]	0.04	− .33	− 11.10	< .001
Social dominance orientation	− 0.16	[− 0.32, − 0.02]	0.08	− .05	− 2.10	.04
Overall model: *R*^2^ = .41, adjusted *R*^2^ = .41, *F*(4, 1385) = 242.13, *p* < .001						

b) LWA	*B*	BCa 95% CI for *B*	*SE B*	β	*t*	*p*
Constant	1.87	[1.57, 2.18]	0.16	−	11.68	< .001
LWA anti-conventionalism	0.99	[0.89, 1.08]	0.05	.67	20.89	< .001
LWA anti-hierarchical aggression	− 0.46	[− 0.55, − 0.36]	0.05	− .30	− 10.16	< .001
LWA top-down censorship	0.05	[− 0.03, 0.12]	0.04	.03	1.30	.19
Overall model: *R*^2^ = .29, adjusted *R*^2^ = .29, *F*(3, 1386) = 192.39, *p* < .001						

*N* = 1390. BCa 95% CI = bias-corrected and accelerated bootstrap 95% confidence interval for the regression coefficient *B* based on 1000 bootstrap samples. TABS = Transgender Attitudes and Beliefs Scale. RWA = right-wing authoritarianism. LWA = left-wing authoritarianism. For the TABS, lower values represent the stronger belief in binary/fixed sex/gender and higher values represent the stronger belief in nonbinary/changeable sex/gender (cf. Kanamori et al., 2017). Higher agreement with the three RWA facets, social dominance orientation, and the three LWA facets is represented by higher values of these variables

While not preregistered, we additionally carried out analogous analyses with the single item on sex/gender belief as the dependent variable (for the results, see Table S3 in the supplementary materials). The respective logistic regression analyses yielded largely parallel results to those obtained with the TABS as the dependent variable; however, RWA authoritarian submission was associated with neither sex/gender belief in this model. Noteworthy, there were no indications of multicollinearity in any of the reported multiple regression models, as all variance inflation factors were < 2.14 and all tolerance parameters were > .46 (cf. Thompson et al., 2017).

In the same way as in Study 1, we also examined the relationships between sex/gender belief and the two types of authoritarianism aspects (i.e., RWA conventionalism and LWA anti-conventionalism) over and above all assessed sociodemographic variables displayed in Table S2. Again, these additional analyses were not preregistered. We regressed sex/gender belief measured with the TABS on RWA conventionalism and the covariates (i.e., RWA authoritarian submission, RWA authoritarian aggression, social dominance orientation, and the sociodemographic variables) in a multiple regression model. We found that a more pronounced binary/fixed sex/gender belief was still related to higher RWA conventionalism (β =  − .37, *p* < .001). A more pronounced binary/fixed sex/gender belief was also still associated with higher RWA conventionalism when the single-item measure of sex/gender belief was regressed on the same variables in a logistic regression model (*B* =  − 1.08, *p* < .001, *OR* = 0.34). Moreover, the nonbinary/changeable sex/gender belief was still related to higher LWA anti-conventionalism when LWA anti-hierarchical aggression, LWA top-down censorship, and the sociodemographic variables were entered in multiple or logistic regression models. This was true when sex/gender belief was regressed as measured by the TABS (β = .32, *p* < .001) as well as the single item measure (*B* = 0.80, *p* < .001, *OR* = 2.22).

## Discussion

Study 2 replicated the findings of Study 1 in a different country: Higher agreement with the binary/fixed sex/gender belief was related to higher RWA conventionalism while higher agreement with the nonbinary/changeable sex/gender belief was related to higher LWA anti-conventionalism. Again, these relationships held over and above the participants’ sociodemographic differences. Moreover, the respective sex/gender beliefs were related to RWA conventionalism and LWA anti-conventionalism independently of their associated other authoritarian aspects (i.e., RWA authoritarian submission, RWA authoritarian aggression, social dominance orientation, LWA anti-hierarchical aggression, and LWA top-down censorship) and also more strongly to (anti-)conventionalism as these other authoritarian aspects. In sum, the results correspond to our preregistered predictions. An interesting side finding that may instigate future research was that LWA anti-hierarchical aggression was correlated with the nonbinary/changeable sex/gender belief; however, when the other LWA aspects were factored out, it was associated with the binary/fixed sex/gender belief. In addition, the results of the regression analyses suggest that RWA authoritarian aggression and LWA anti-hierarchical aggression may play a unique role in terms of sex/gender beliefs. In contrast, the remaining covariates in the regression models—RWA authoritarian submission, social dominance orientation, and LWA top-down censorship—may not be of particular importance for an individual’s sex/gender belief as the respective relationships were statistically non-significant or of rather small size.

## General Discussion

### The Present Findings

In line with our preregistered predictions, in two studies we found that sex/gender beliefs were associated to both RWA conventionalism and LWA anti-conventionalism, independent of the other aspects of authoritarianism, sociodemographic variables, and whether sex/gender belief was measured on a spectrum or as a dichotomy. These relationships were strong (i.e., *r*s ≥ .30) according to recent classifications of effect sizes (Funder & Ozer, 2019; Gignac & Szodorai, 2016). Conceptually replicating previous research (e.g., Ching et al., 2020; Makwana et al., 2018; Perez-Arche & Miller, 2021; Schudson & van Anders, 2022), we found that the binary/fixed sex/gender belief was associated with RWA conventionalism. In addition to this known relationship, we also found the nonbinary/changeable sex/gender belief to be associated with LWA anti-conventionalism. Thus, both opposing beliefs of sex and gender were related to rigid and restrictive authoritarianism, particularly to the inclination to absolutize conservative or progressive moral values, absolutely reject and discredit other views, and impose the conventional or anti-conventional ideological concepts on everyone in society (cf. Altemeyer, 2006; Costello et al., 2022; Duckitt et al., 2010; Osborne et al., 2023). As LWA anti-conventionalism was examined regarding sex/gender beliefs for the first time, the present two studies provide a more balanced view of the matter than was previously the case.

Our findings may be interpreted such that the rough differentiation of two sex/gender beliefs reflects two directions in which one can principally think about sex and gender: conventionally or contrary to the convention/anti-conventionally (cf. Hatch et al., 2022; Konopka et al., 2020; McGuire et al., 2019). This trivial classification can be made without any considerations of authoritarianism. The relationships between sex/gender beliefs and authoritarianism that we found in the present research are probably due to the fact that, in addition to the many individuals relatively low in authoritarianism, the groups of conventionally or anti-conventionally oriented individuals also include those who are relatively high in the respective authoritarianism.

Another finding of Study 2 was that RWA conventionalism and LWA anti-conventionalism were stronger predictors of the respective sex/gender beliefs than any of the other authoritarianism aspects. This finding is in line with our expectations, as we assumed a strong conceptual proximity between a conventional sex/gender belief and a fundamentally conservative-conventional attitude (Konopka et al., 2020; Perez-Arche & Miller, 2021), and correspondingly, between an anti-conventional sex/gender belief and a fundamentally progressive-anti-conventional attitude. Accordingly, the respective associations with sex/gender beliefs should also be primarily observable when these fundamental attitudes are represented in an authoritarian way. It is less clear, however, why other aspects of authoritarianism were also related to sex/gender beliefs, over and above authoritarian (anti-)conventionalism—as the multiple regression models in Study 2 found. Based on the data available to date, it seems premature to speculate on the reasons for these found relationships that are independent of authoritarian (anti-)conventionalism. The expected relationships between (anti-)conventionalism and sex/gender beliefs are reasonable on a theoretical basis and were replicated in the present research in Study 2; however, this is not the case for the other aspects of authoritarianism, which were not in the focus of this study, which were only assessed in Study 2, and for which no preregistered predictions were made. Therefore, we refer the respective open questions of replicability and explanation to future research.

One crucial point in terms of the interpretation is that the present findings cannot mean that sex/gender beliefs are authoritarian *per se*. Such a notion would imply that there are only authoritarian individuals in society as both opposing sex/gender beliefs were substantially related to authoritarian attitudes. This obviously absurd conclusion would be based on the incorrect interpretation of correlation coefficients—even a strong correlation does neither allow for a specific belief to be called authoritarian in an absolute sense nor does it permit the conclusion that a sex/gender belief and an authoritarian aspect are identical constructs under different labels (cf. Borsboom et al., 2004). Rather, our findings implicate that each sex/gender belief is related to a relative agreement with RWA conventionalism and LWA anti-conventionalism on a spectrum from low to high.

According to social science researchers (e.g., Irving, 2008; Spencer et al., 2021), there are systemic power structures in societies such as the USA or UK oppressing nontraditional gender identities. Based on such an assumption, one could challenge the comparability of the nonbinary/changeable sex/gender belief with the binary/fixed sex/gender belief with regard to their authoritarian charge. While the conventional sex/gender belief has historically been embedded in these cultures for a long time, the anti-conventional sex/gender belief could have been downplayed and marginalized. We have the perspective, however, that historical-interpretative approaches on a sociological level or the normative framework of social justice research has no immediate relevance for our present empirical research which, therefore, should not be interpreted in that way. The focus of our measures and analyses was at the level of already acquired factual views (i.e., of sex/gender and authoritarian attitudes) and how they are empirically related at a certain time. Our research is not about which beliefs or attitudes had or have the better chance of being adopted by the majority of individuals in a given culture and how the corresponding societal developments are associated with objective or perceived oppression. Rather, we directly captured the participants’ interrelated views which they hold at the given moment and which may be the result of different cultural acquisition processes to that date. It is also worth noting that, at the time of our studies, the belief that there are not only two genders no longer appears to be downplayed, as it was held by many participants rather than a small minority (i.e., 43% in our U.S. sample and 32% in our UK sample).

### Limitations and Future Research

The findings of our two studies provide initial empirical evidence to overcome the one-sided state of the research literature on authoritarianism and sex/gender beliefs. There are, however, still unanswered questions and limits of insight. For instance, we did not investigate the relationships between authoritarianism and sex/gender beliefs in depth by assessing and analyzing potential moderating and mediating variables. Instead, we addressed what Zanna and Fazio (1982) called the first-generation research questions, that is, empirically showing the presence of an association, as this has not yet been undertaken for LWA anti-conventionalism in the context of sex/gender beliefs. Further investigation of the second-generation research questions (i.e., moderation and mediation) should follow next. In this endeavor, it would also be desirable to examine the causal path from authoritarianism (presumed cause) to beliefs (presumed outcome), which we have not done in the present studies. This future research would be necessary for both RWA conventionalism and LWA anti-conventionalism.

A point related to the question of moderation and mediation is whether meaningful subgroups within the different sex/gender beliefs can be differentiated from each other. Possibly, some individuals are using authoritarian methods to actually live out improper motives (e.g., living out hatred or fantasies of power; in this regard, see the research that found associations between left-/right-wing authoritarianism and narcissism as well as psychopathy; Krispenz & Bertrams, 2024; Roy et al., 2021; Zeigler-Hill et al., 2021). In addition to such individuals, however, there is probably a larger number of individuals with a binary/fixed sex/gender belief or a nonbinary/changeable sex/gender belief who do not pursue such purposes. In further studies, the relationships between authoritarianism and sex/gender beliefs that we found could possibly be traced back to so-called dark personality traits (e.g., narcissism and psychopathy) and in this way be understood more accurately. The present two studies, however, do not allow for such analyses.

Furthermore, while the instrument we applied to measure sex/gender beliefs (Kanamori et al., 2017) is standardized and common, it does not differentiate between aspects of these sex/gender beliefs (e.g., such relating to biological sex and those addressing variations in social gender). It may be useful in future research to differentiate the individual components under the umbrella of sex/gender beliefs in more detail regarding their relationships with authoritarianism. In addition, as we have already noted above, further research on sex/gender beliefs and authoritarianism should also address the other aspects of authoritarianism apart from (anti-)conventionalism.

A particular important alley for future research may be to what extent the relationships we found on the opposing sides of sex/gender belief and authoritarianism represent a symmetry. As we outlined in the introduction, there is an ongoing debate and research about the extent to which the types of authoritarianism on the different political sides mirror each other or not (Conway et al., 2018; Costello et al., 2022; Nilsson, 2024; Osborne et al., 2023). As we have pointed out, the standardized RWA and LWA measures that we used were not developed in tandem and are not strictly symmetrical in terms of their content and connotations. For instance, in the measure of RWA conventionalism dissenting ways of living and behaving are belittled as “radical and sinful” (Duckitt et al., 2010) whereas in the measure of LWA anti-conventionalism even direct insults against ideological opponents are contained (e.g., “All political conservatives are fools,” Costello et al., 2022). Therefore, it is yet unclear whether, just due to measurement reasons, other relevant variables (e.g., aggressiveness) are incorporated to different extents in the relationship between RWA conventionalism and the binary/fixed sex/gender belief versus the relationship between LWA anti-conventionalism and the nonbinary/changeable sex/gender belief. Based only on the data of the present two studies, respective interpretations are not possible.

Finally, we would like to stress that our findings only apply to the USA and the UK, but sex/gender issues are currently causing controversy in many cultures. Therefore, in addition to the inherent value of replication, the relationships found in our studies should also be investigated in other cultures/countries. During our research, we found many incidents in different countries that are relevant to the question at hand (e.g., for Germany: Jurjaks, 2024; Upton, 2022); thus, we would expect similar results.

## Supplementary Information

Below is the link to the electronic supplementary material.

Supplementary file1 (PDF 562 kb)

## Data Availability

Prior to the data collection, the hypothesis and analysis plan for the current research were preregistered at https://aspredicted.org. The pre-registrations, full datasets, and complete materials of the current studies are available at https://researchbox.org/2039.
