# Supplementary material for: Sex/Gender Beliefs are Strongly Related to Either Right-Wing Authoritarian Conventionalism or Left-Wing Authoritarian Anti-Conventionalism
Source: Arch Sex Behav. 2026 Feb 18;55(2):809–22. doi: 10.1007/s10508-025-03371-4 (PMC13048938; doi:10.1007/s10508-025-03371-4)
Supplement: Supplementary file 1 — Supplementary file1 (PDF 562 kb) [file 10508_2025_3371_MOESM1_ESM.pdf]

Sex/Gender Beliefs are Strongly Related to Either Right-Wing Authoritarian Conventionalism or Left-Wing Authoritarian Anti-Conventionalism

Supplementary Material

Table S1

Sample Demographics for Study 1 (U.S. Sample)

	N	*M*	*SD*	%
Age (years) [a]	1,429	44.58	15.26	
Gender [b]				
Female	717			50.1
Male	690			48.2
Nonbinary	23			1.6
Transgender	13			0.9
Other	1			0.1
Sexual orientation				
Bisexual	151			10.6
Heterosexual, straight	1,179			82.4
Homosexual, gay/lesbian	68			4.8
Other	33			2.3
Marital status				
Single (never married)	484			33.8
Married (first marriage)	519			36.3
Remarried	69			4.8
Separated	19			1.3
Divorced	156			10.9
Widowed	46			3.2
Long-term domestic partner (at least one year)	138			9.6
Political orientation on left-right spectrum [c]	1,431	3.31	1.59	
Political ideology				
Conservative	260			18.2
Leftist	128			8.9
Liberal	620			43.3
Libertarian	61			4.3
Moderate	354			24.7
Populist	8			0.6
Political party identification				
Democratic	663			46.3
Green	9			0.6
Independent	343			24.0
Libertarian	34			2.4
Republican	247			17.3
Socialist	50			3.5
None	68			4.8
Other	17			1.2

Table S1 [*continued*]

	N	*M*	*SD*	%
Ethnicity [b]				
American Indian or Alaska Native	19			1.3
Asian or Asian-American	76			5.3
Black or African-American	187			13.1
Hispanic or Latino	77			5.4
Native Hawaiian or Pacific Islander	2			0.1
White	1,133			79.2
Other	7			0.5
Native language				
English	1,363			95.2
English and another language	47			3.3
Another language	21			1.5
Highest educational level				
Professional degree (JD, MD)	24			1.7
Doctoral degree	21			1.5
Master's degree	185			12.9
Bachelor's degree in college (four-year)	571			39.9
Associate degree in college (two-year)	169			11.8
Some college but no degree	278			19.4
High school graduate	171			11.9
Less than high school degree	9			0.6
Other	3			0.2
Household income [d]				
Less than $10,000	56			3.9
$10,000 to $19,999	85			5.9
$20,000 to $29,999	120			8.4
$30,000 to $39,999	134			9.4
$40,000 to $49,999	138			9.6
$50,000 to $59,999	154			10.8
$60,000 to $69,999	108			7.5
$70,000 to $79,999	105			7.3
$80,000 to $89,999	84			5.9
$90,000 to $99,999	79			5.5
$100,000 to $149,999	210			14.7
$150,000 or more	158			11.0

Note. [a] Two indications were omitted due to impossible values. [b] The participants could select multiple choices. [c] Selection on a seven-point scale from *extremely left-wing* (1) to *extremely right-wing* (7). [d] Household income includes the entire household income (in previous year) before taxes.

Table S2

Sample Demographics for Study 2 (UK Sample)

	N	*M*	*SD*	%
Age (years)	1,390	46.92	15.40	
Gender [a]				
Female	708			50.9
Male	676			48.6
Nonbinary	6			0.4
Transgender	5			0.4
Other	2			0.1
Sexual orientation				
Bisexual	79			5.7
Heterosexual, straight	1,263			90.9
Homosexual, gay/lesbian	34			2.4
Other	14			1.0
Marital status				
Single (never married)	431			31.0
Married (first marriage)	520			37.4
Remarried	73			5.3
Separated	21			1.5
Divorced	91			6.5
Widowed	28			2.0
Long-term domestic partner (at least one year)	226			16.3
Political orientation on left-right spectrum [b]	1,390	3.63	1.18	
Political ideology				
Conservative	273			19.6
Leftist	247			17.8
Liberal	428			30.8
Libertarian	40			2.9
Moderate	388			27.9
Populist	14			1.0
Political party identification				
Conservative and Unionist Party	156			11.2
Green Party of England and Wales	142			10.2
Labour Party	400			28.8
Liberal Democrat	160			11.5
Reform UK	140			10.1
Scottish National Party	41			2.9
None	281			20.2
Other	70			5.0

Table S2 [*continued*]

	N	*M*	*SD*	%
Ethnicity [a]				
Asian or Asian British	102			7.3
Black, Black British, Caribbean, or African	49			3.5
White	1,208			86.9
Other	36			2.6
Native language				
English	1,261			90.7
English and another language	80			5.8
Another language	49			3.5
Highest educational level				
Doctoral degree	38			2.7
Master’s degree	197			14.2
Bachelor’s degree	550			39.6
A-Levels or equivalent	351			25.3
General Certificate of Education or equivalent	213			15.3
Secondary education not finished	9			0.6
Other	32			2.3
Household income [c]				
Less than £10,000	63			4.5
£10,000 to £19,999	159			11.4
£20,000 to £29,999	284			20.4
£30,000 to £39,999	209			15.0
£40,000 to £49,999	194			14.0
£50,000 to £59,999	143			10.3
£60,000 to £69,999	115			8.3
£70,000 to £79,999	66			4.7
£80,000 to £89,999	49			3.5
£90,000 to £99,999	41			2.9
£100,000 to £149,999	52			3.7
£150,000 or more	15			1.1

Note. [a] The participants could select multiple choices. [b] Selection on a seven-point scale from *extremely left-wing* (1) to *extremely right-wing* (7). [c] Household income includes the entire household income (in previous year) before taxes.

Table S3

Logistic Regression Analysis Regressing Sex/Gender Beliefs (Measured With the Dichotomous Single Item) in Study 2 (UK Sample)

a) RWA and social dominance orientation	*B*	BCa 95% CI for *B*	*SE B*	*OR*	Wald	*df*	*p*
Constant	5.21	[3.81, 6.69]	0.72	183.10	52.48	1	< .001
RWA conventionalism	−0.93	[−1.15, −0.74]	0.11	0.39	77.39	1	< .001
RWA authoritarian submission	0.04	[−0.14, 0.22]	0.09	1.04	0.15	1	.70
RWA authoritarian aggression	−0.38	[−0.53, −0.24]	0.08	0.68	23.59	1	< .001
Social dominance orientation	−0.42	[−0.75, −0.06]	0.17	0.66	5.81	1	.02
Overall model: χ^2 (4, N = 1,390) = 382.03, Cox and Snell R^2 = .24, Nagelkerke R^2 = .34, p < .001							

b) LWA	*B*	BCa 95% CI for *B*	*SE B*	*OR*	Wald	*df*	*p*
Constant	−4.04	[−4.79, −3.46]	0.35	0.02	135.58	1	< .001
LWA anti-conventionalism	1.33	[1.13, 1.58]	0.10	3.79	174.13	1	< .001
LWA anti-hierarchical aggression	−0.57	[−0.76, −0.40]	0.09	0.57	42.01	1	< .001
LWA top-down censorship	−0.01	[−0.15, 0.15]	0.07	0.99	0.02	1	.89
Overall model: χ^2 (3, N = 1,390) = 278.93, Cox and Snell R^2 = .18, Nagelkerke R^2 = .26, p < .001							

Note. N = 1,390. BCa 95% CI = bias-corrected and accelerated bootstrap 95% confidence interval for the regression coefficient B based on 1,000 bootstrap samples. OR = odds ratio. RWA = right-wing authoritarianism. LWA = left-wing authoritarianism. For the single item on sex/gender belief, 0 represents the stronger belief in binary/fixed sex/gender and 1 represents the stronger belief in nonbinary/changeable sex/gender. Higher agreement with the three RWA facets, social dominance orientation, and the three LWA facets is represented by higher values of these variables.

Figure S1

Scatter Plots for the Relationships Between RWA Conventionalism/LWA Anti-Conventionalism and Sex/Gender Belief (Measured With TABS) in Study 1 (U.S. Sample) and Study 2 (UK Sample)

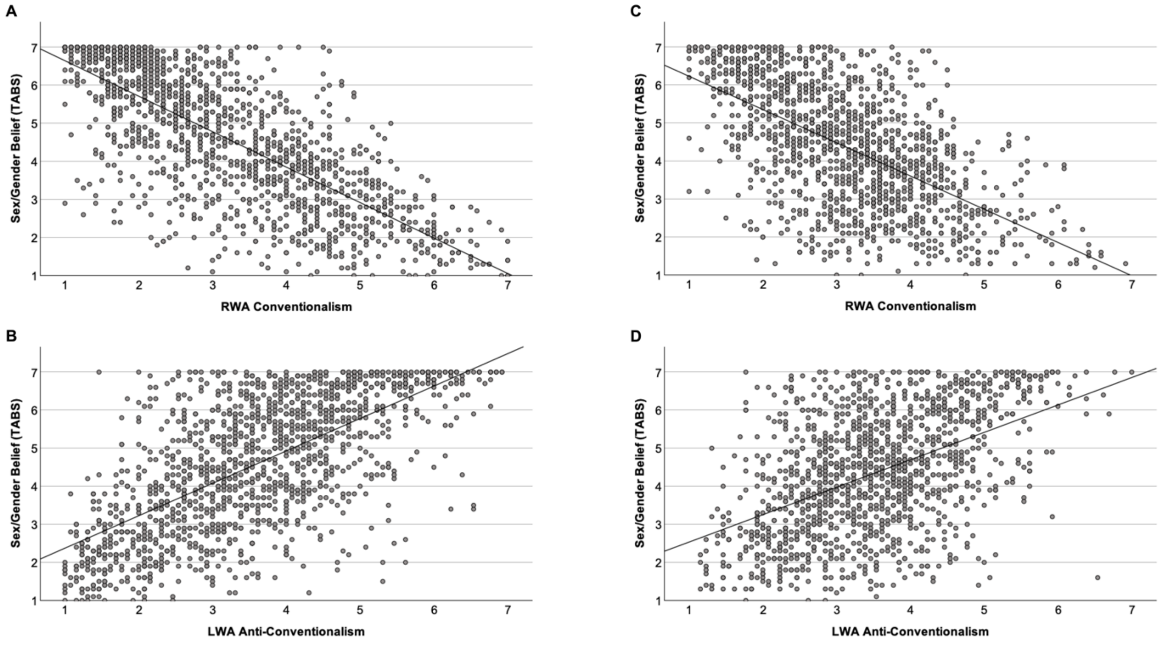

Note. A and B: Data from Study 1 (N = 1,431). C and D: Data from Study 2 (N = 1,390). Each dot represents an individual participant, the regression lines are also displayed. RWA = right-wing authoritarianism. LWA = left-wing authoritarianism. TABS = Transgender Attitudes and Beliefs Scale. The total scores of the psychometric scales were obtained by averaging the responses to the scale items. Higher agreement with RWA conventionalism and LWA anti-conventionalism is represented by higher values of these variables. For the TABS, lower values represent the stronger belief in binary/fixed sex/gender and higher values represent the stronger belief in nonbinary/changeable sex/gender.
